# Comparative Analysis of Mechanical Properties and Metal-Ceramic Bond Strength of Co-Cr Dental Alloy Fabricated by Different Manufacturing Processes

**DOI:** 10.3390/ma11101801

**Published:** 2018-09-22

**Authors:** Xingting Han, Tomofumi Sawada, Christine Schille, Ernst Schweizer, Lutz Scheideler, Jürgen Geis-Gerstorfer, Frank Rupp, Sebastian Spintzyk

**Affiliations:** 1 Section Medical Materials Science and Technology, University Hospital Tübingen, Osianderstr. 2-8, Tübingen 72076, Germany; xingting.han@student.uni-tuebingen.de (X.H.); Tomofumi.Sawada@med.uni-tuebingen.de (T.S.); Christine.Schille@med.uni-tuebingen.de (C.S.); ernst.schweizer@med.uni-tuebingen.de (E.S.); lutz.scheideler@med.uni-tuebingen.de (L.S.); juergen.geis-gerstorfer@med.uni-tuebingen.de (J.G.-G.); Frank.Rupp@med.uni-tuebingen.de (F.R.); 2 Department of Biomedical Engineering, Iwate Medical University, 2-1-1 Nishitokuta, Yahaba-cho, Shiwa-gun, Iwate 028-3694, Japan

**Keywords:** Cobalt-chromium (Co-Cr) alloy, additive manufacturing, selective laser melting (SLM), milling, casting, mechanical properties, bond strength, microstructure, dental prostheses

## Abstract

Cobalt-chromium (Co-Cr) alloy is a widely used base material for dental fixed prostheses. These restorations can be produced through casting technique, subtractive or additive manufacturing technologies. However, limited information is available regarding the influence of manufacturing techniques on the properties of Co-Cr alloy since most studies used different chemical compositions of Co-Cr alloy for different manufacturing methods. This study compares the mechanical properties, metal-ceramic bond strength, and microstructures of specimens produced by casting, milling, and selective laser melting (SLM) from one single Co-Cr alloy composition. The mechanical properties of the alloy were investigated by tensile and Vickers hardness tests, and metal-ceramic bond strength was determined by three-point bending. Scanning electron microscopy (SEM) with backscattered electron (BSE) images and optical microphotographs were used to analyze the surface microstructures. Compared with the casting and milling techniques, SLM Co-Cr alloy specimens indicated enhanced mechanical properties and comparable metal-ceramic bond strength. Besides, the microstructures of the SLM specimens showed finer grains with more second phase particles than the casting and milling specimens. The results of our study indicate that SLM might be superior to traditional techniques for the manufacturing of fixed dental restorations.

## 1. Introduction

Metal-ceramic prostheses, which are known as the porcelain-fused-to-metal (PFM) restorations, have been widely used in fixed dental prosthodontics because of the proper mechanical and aesthetic properties [1,2]. Among many different noble and base dental alloys, cobalt-chromium (Co-Cr) is one of the common alloys to fabricate the substrate of metal-ceramic restorations because of the low cost, high mechanical strength, and good corrosion resistance [3,4].

Traditionally, Co-Cr substrates are fabricated by the lost-wax casting technique, which is still the dominant method in dental metal processing. However, this technique requires a lot of time and certain skills for dental technicians and often results in pores and defects in the interior of the alloy [5,6]. Recently, computer-aided design and computer-aided manufacturing (CAD-CAM) technology has been introduced to fabricate the substrates. This technology can be divided into two main categories: Subtractive manufacturing processes like milling, and additive manufacturing processes like selective laser melting (SLM). Milling usually uses tools, such as saws, lathes, grinders, and drill presses to mechanically cut the block to the desired geometry, are controlled by a computer program. Milling can reduce flaws and pores which may be caused by the casting process because the Co-Cr blanks are made under high industrial standards, and the sample precision is related to the cutting performance [7]. The disadvantages of milling are the waste of materials and limited potential for complex products compared with casting and SLM [8]. SLM is a laser-based additive manufacturing technology, which is widely used in medical fields in recent years, especially for dental restorations like customized crowns, bridges, abutments, and screw-retained restorations [9,10,11,12,13]. SLM produces the metal substrate by fusing metal powder in layers without much porosity. This technique uses a focused high-power laser beam and results in products of nearly 100% density. The laser could also be the key to harden metals and ceramics [14]. Furthermore, objects with complex geometries are achievable with a high-dimensional accuracy [15,16,17,18]. Compared to casting and milling processes, SLM reduces the probability of operator errors, minimizes defects and wastes almost no material since the remaining powder can be used further [19]. 

For the longevity of clinical use, PFM restorations have to comply with certain requirements, including mechanical properties, metal-ceramic bond strength, biocompatibility, and corrosion resistance [20]. While SLM is a promising technique for the fabrication of prostheses, the lack of investigations of mechanical properties and metal-ceramic bond strength of SLM-processed restorations is still a significant drawback [21]. These properties must be further investigated to ensure that the quality of SLM prostheses is at least equal to those produced by conventional techniques [22]. Under a variety of testing methods, the tensile test and three-point bending test (Schwickerath crack initiation test), which were promulgated in ISO 9693-1: 2012 and ISO 22674: 2016 for dental restorations, are the most common standards to evaluate the mechanical properties and metal-ceramic bond strength in dental research [23,24]. The mechanical properties of the alloy can be influenced by porosity, grain size, and second-phase particles [25,26]. Metal-ceramic bond strength is related to Young’s (elastic) modulus, chemical bonding, mechanical interlocking, compressive bonding, and van der Waals forces, among those, chemical bonding dominates [6,27,28,29]. The chemical bonding can be changed when an oxide layer forms on the surface. The oxides are formed while wetting the alloy with veneering ceramic. A thin oxide layer can be eliminated during ceramic firing and gain an excellent bond strength. Whereas, an excessively thick oxide layer may allow more oxide from the ceramic to participate in the chemical reaction and lead to a broader interaction region, and thus weaken the chemical bonding [30,31,32]. Besides, it is essential to characterize microstructures due to their possible relationship with mechanical properties and the metal-ceramic bond strength, using methods like scanning electron microscopy (SEM) with backscattered electron (BSE) images, and optical microscopy [33]. Normally, SLM has a finer grain size compared to conventional casting technique due to the rapid cooling rate during the building process, which permits the production of bulk objects with very fine microstructures and enhanced mechanical properties [34].

Some studies have already compared the characteristics of Co-Cr alloys fabricated by casting, milling and SLM [6,26,35,36]. However, in their reports, different chemical compositions of the alloys for different manufacturing processes were used, which may be due to the limitations of techniques and materials [6,36,37,38,39]. Recently, only one publication has examined the shear bond strength of a Co-Cr alloy with the same chemical composition manufactured by different processes [5]. In the study of Antanasova et al., no significant difference in shear bond strength of Co-Cr alloy by casting, milling, and SLM was found. This result is probably because, after the firing process, the samples by different manufacturing methods had similar oxidation properties, resulting in similar chemical bonds. Besides, the similar Young’s modulus among different groups also plays an essential role in the bond strength due to the same rigidity to resist bending and delamination [5]. To estimate the reliability of the real effect of manufacturing techniques on alloy properties and eliminate the influence of chemical composition, we prepared the specimens using a Co-Cr alloy with entirely the same composition throughout casting, milling and SLM groups. The null hypothesis is that different manufacturing processes will not influence the mechanical properties of Co-Cr alloy and metal-ceramic bond strength. 

## 2. Materials and Methods

**Figure 1:**
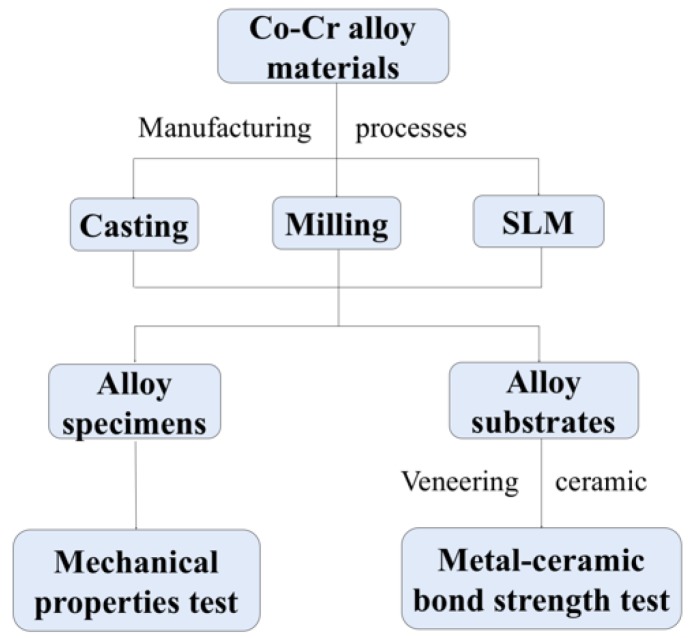
The flow chart of the experiment.

The flow chart of the experiment is presented in Figure 1. Co-Cr alloy specimens and substrates for mechanical properties test and metal-ceramic bond strength test were fabricated by three manufacturing methods. The groups and materials used in this study are presented in Table 1.

### 2.1. Specimen Preparation and Characterization of the Surface

#### 2.1.1. Specimens Preparation for Mechanical Properties Test

**Figure 2:**
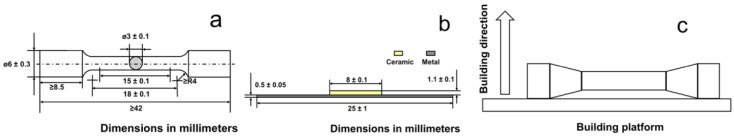
Schematic drawing of the testing specimens with specified dimensions for mechanical properties test (**a**) and metal-ceramic bond strength test (**b**), according to ISO 22674: 2016 and ISO 9693-1: 2012. (**c**) The building direction of the SLM specimens.

For the mechanical properties test, metal specimens were fabricated by casting, milling, and SLM manufacturing processes (*n* = 6 per group). The dumbbell-shaped dimension of the specimens according to ISO 22674: 2016 is presented in Figure 2a [24]. 

In the casting group, Co-Cr samples were manufactured by the conventional lost wax technique. Wax rods were cut into a dumbbell-shaped dimension as a template and mounted in a silicone ring. After modeling with the inlay casting wax, samples were invested in phosphate-bonded investment (Onyx, Heraeus-Kulzer, Hanau, Germany). Then, the rings were put into a furnace (Mihm + Voght, Stutensee, Germany) to evaporate the wax. The casting was accomplished using the Co-Cr alloy ingots (Remanium Star, Dentaurum, Ispringen, Germany) in a casting device (Combilabor CL-I 95, Heraeus-Kulzer, Hanau, Germany) according to the manufacturer’s instructions. For casting samples, there was no post-production heat treatment, after cooling at room temperature, the samples were divested, cleaned, and sandblasted with 125 μm Al_2_O_3_ particles under 0.4 MPa pressure (Cemat NT4, Wassermann, Hamburg, Germany). 

For the milling group, the samples were designed by CAD software (Exocad Dental Cad, Exocad, Darmstadt, Germany), and then exported to CAM software (Sum3d Dental, CIM system, Cinisello Balsamo, Italy). The specimens were milled from a prefabricated Co-Cr alloy blank (98.4 mm in diameter and 12 mm in thickness, Remanium Star MD, Dentaurum, Ispringen, Germany) by a milling machine (550i, imes icore, Eiterfeld, Germany) according to the manufacturer’s instructions. Then the milling specimens were cut out using a cutting disc and sandblasted in the same manner as the casting samples. There was no heat treatment after the milling process. 

For the SLM group, the samples were also designed by CAD software (Dassault Systemes, Waltham, MA, USA) and transferred to an SLM device (Mlab, Concept Laser, Lichtenfels, Germany) equipped with a 100 W fiber laser. The size of the Co-Cr powder (Remanium Star CL, Dentaurum, Ispringen, Germany) was 10–40 μm. SLM manufacturing parameters were based on the standard process recommended by the manufacturer, the building layer thickness was 25 mm, scan speed was 7 m/s, manufacturing speed was 5 cm^3^/h, Yb-fiber laser power was 100 W, and the longitudinal axes of the specimens was parallel to the building platform (Figure 2c). After fabricating, the SLM samples were subjected to post-build heat treatment in a furnace (Nabertherm N 41 H, Lilienthal, Germany) to remove the residual stress arising from the local laser melting and to tailor the microstructure according to the manufacturer’s recommendation. The heat treatment was under an argon atmosphere at 1150 °C for 1 h, then cooling down to furnace temperature (200 °C). After cooling, the samples were removed from the furnace and left to completely cool at room temperature. A dental hand piece (K-POWERgrip, KaVo, Warthausen, Germany) was used for the removal of the supporting structures. After all, the specimens were cleaned and sandblasted in the same manner as the casted samples.

#### 2.1.2. Specimens Preparation for Metal-Ceramic Bond Strength Test

The alloy substrates of the metal-ceramic specimens (*n* = 109; 29 for the casting group, 40 for the milling group and 40 for the SLM group) were prepared using the same devices and processes. The dimensions of the substrates were (25 ± 1) mm × (3 ± 0.1) mm × (0.5 ± 0.05) mm according to ISO 9693-1: 2012 [23]. The opaque ceramic was applied over a length of (8 ± 0.1) mm symmetrically on one 3 mm-wide side of each specimen on the substrate surface (Figure 2b). After the manufacturing processes, the surface of the alloy substrates was sandblasted with 125 μm Al_2_O_3_ particles, then cleaned with steam and dried with compressed air. 

#### 2.1.3. Surface Characterization

Before firing the ceramic, four metal substrates were selected randomly from each group to determine the surface roughness. The average roughness (R_a_) was measured by profilometry (Perthometer Concept S6P, Mahr, Göttingen, Germany) over the length of 5.6 mm with 3 single profiles per sample, providing 12 readings totally per group.

#### 2.1.4. Application of the Veneering Ceramic

Ceramic powder and liquid (Ceramotion Me, Dentaurum, Germany) were mixed in a vacuum-vibrator and applied in a metal jig. The opaque and dentine firing steps were applied twice on each metal stripe, respectively, followed by the glaze firing step once. The firing processes were performed in a furnace (DEKEMA 3001, Freilassing, Germany) according to the manufacturer’s instructions (Table 2).

### 2.2. Mechanical Properties Test and Microstructure Analysis

Mechanical properties were divided into two parts, tensile and Vickers hardness test. The tensile test was performed on the alloy specimen (Figure 2a) according to ISO 22674: 2016 using a universal testing machine Z020 (Zwick, Ulm, Germany) at a crosshead speed of 2 mm/min until a fracture occurred [24]. The 0.2% yield strength (R_p0.2_), percentage elongation at fracture, ultimate tensile strength (R_m_), and Young’s modulus were measured for the tensile test. A Vickers hardness-testing machine (HV 10, Wolpert, Bretzfeld, Germany) was used for hardness testing on the edge area of the dumbbell-shaped specimens, where they were clamped by the specimen grips in the tensile test. A load of 10 kg was applied for 15 s of dwelling time. 

For the microstructural analysis, three specimens per group were cut along the longitudinal axes to expose the cross-section and embedded in epoxy resin (Orthocryl, Dentaurum, Ispringen, Germany). After polymerization, the cross-section of the specimens was polished with silicon carbide abrasive paper up to P2500, followed by fine polishing using a medium-hard silk gamma cloth with water-based 3 μm polycrystalline diamond paste (Metadi II, Buehler, Esslingen, Germany) and suspension (Dialub SW, Buehler, Esslingen, Germany). The polished surfaces were etched with 37% HCl with a few drops of H_2_O_2_ until the surface appeared slightly dull. The specimens were rinsed with plenty of water and finally dried with compressed air. The former clamped area by the specimen grips in the tensile test was observed with an SEM/BSE machine (LEO 1430, Zeiss, Oberkochen, Germany, magnification 500×, 2000×, and 5000×). The grain size could be calculated roughly by software (Measure, Datinf, Tübingen, Germany) with the scaling bar inserted. After defining the length of the scaling bar, the ratio (real length/represented length) was used to measure the widest and narrowest area of the grain. The fractured surface of the specimen was also observed by SEM (LEO 1430, Zeiss, Oberkochen, Germany, magnification 65× and 1000×).

### 2.3. Bond Strength Test and Microstructure Analysis

The metal-ceramic bond strength was measured by the three-point bending test with a universal testing machine Z010 (Zwick, Ulm, Germany). Specimens (Figure 2b) were placed in the bending apparatus (20 mm distance between the supports and 1 mm radius of the bending piston) with the ceramic positioned opposite to the applied force symmetrically. A crosshead speed of 1.5 mm ± 0.5 mm/min was applied until cracking occurred, and the respective failure force (F_fail_) was recorded for each specimen. The calculation of bond strength (τ_b_) is given by the following equation (ISO 9693: 2012 [23]):

τ_b_ = F_fail_(A × d_m_^2^ + B × d_m_ + C), (1)

where A, B, and C are correction factors, which are calculated by the Young’s modulus of the tensile test, and d_m_ is the thickness of the specimens.

After the three-point bending test, first, the crack between metal and ceramic was analyzed by SEM laterally (magnification 1000×). Subsequently, the metal-ceramic specimens were debonded manually to analyze the remaining ceramic at the alloy surface. The former contact area of the alloy substrate was characterized with SEM (magnification 1000×) and energy dispersive X-Ray spectroscopy (EDX). An optical microscope (M400, Wild Heerbrugg AG, Heerbrugg, Switzerland, magnification 16×) and a software (Measure, Datinf, Tübingen, Germany) were used to analyze the failure mode and area fraction of adhering ceramic after debonding. The failure mode can be classified into three types: Adhesive, less than 20% of the bonding area is covered by remained ceramic in the Co-Cr substrate surface; mixed, ceramic remained more than 20%, but less than 80%, and cohesive was more than 80% of the alloy surface covered by the remaining ceramic.

### 2.4. Statistical Analysis

SPSS Version 21 (SPSS INC, Chicago, IL, USA) was used for analyzing the data. Shapiro–Wilk and Levene tests were applied to assess the assumptions of data normality and the homogeneity of variances. One-way analysis of variance (ANOVA) was used for data of roughness, elongation, Young’s modulus, and area fraction of adhered ceramic, followed by a Tukey post-hoc test (α = 0.05). The R_p0.2_, R_m_, Vickers hardness, and bond strength were analyzed by Kruskal-Wallis analysis (α = 0.05) for the disobedience of the data normality or homogeneity of variances.

## 3. Results

### 3.1. Surface Characteristics of the Metal Substrate

The R_a_ values for each group are presented in Table 3. The SLM group showed the highest R_a_ value of all the three groups (*p* < 0.05), while there was no statistical difference between the casting and milling groups. 

### 3.2. Mechanical Properties and Microstructure of Co-Cr Alloy Specimens

As shown in Table 4, the R_p0.2_, R_m_ and Vickers hardness of the SLM group were the highest among the three groups (*p* < 0.05), while the casting group showed the lowest values (*p* < 0.05). The SLM group also indicated a higher elongation at fracture than the milling group (*p* < 0.05). For Young’s modulus, the milling group demonstrated the highest value (*p* < 0.05). All groups exceeded the minimum required of R_p0.2_ (80–500 MPa) and Young’s modulus (150 GPa) for all types in ISO 22674: 2016 (type 0, small veneered one-surface inlays, veneered crowns; type 1, veneered or un-veneered one-surface inlays, veneered crowns; type 2, crowns or inlays without restriction on the number of surfaces; type 3, multiple unit fixed prostheses; type 4, removable partial dentures, clasps, thin veneered single crowns, and full arch fixed dental prostheses; type 5, thin removable partial dentures, parts with thin cross-sections, clasps) [24]. For the elongation at fracture, the value of casting and SLM specimens were higher than the ISO requirement for type 2–5 (2–10%), but for milling, the elongation value only beyond type 3–5 (2–5%).

**Figure 3:**
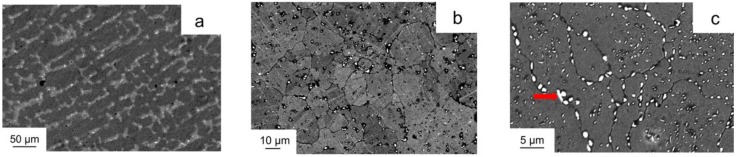
BSE images of Co-Cr alloy specimens: (**a**) casting, magnification 500×; and (**b**) milling, magnification 2000×; (**c**) SLM, magnification 5000×. The red arrow represents the bright blocky precipitate.

Figure 3 shows the BSE images of the cross-section of the Co-Cr specimens. The casting group displayed a typical inhomogeneous dendritic solidification microstructure consisting of dendritic areas and interdendritic areas (Figure 3a). The grain size of casting specimen is about 20–100 μm. In contrast, the milling group showed a homogenous microstructure with a grain size of five–40 μm (Figure 3b). A much finer and non-equilibrium structure could be seen in SLM groups with a grain size of 2–20 μm (Figure 3c). In the milling and SLM groups, some second phase particles were randomly distributed in the interior and at the boundaries of the grains, especially for the SLM samples. Besides, in the SLM group, some bright blocky precipitate was dispersed randomly at the grain boundaries.

**Figure 4:**
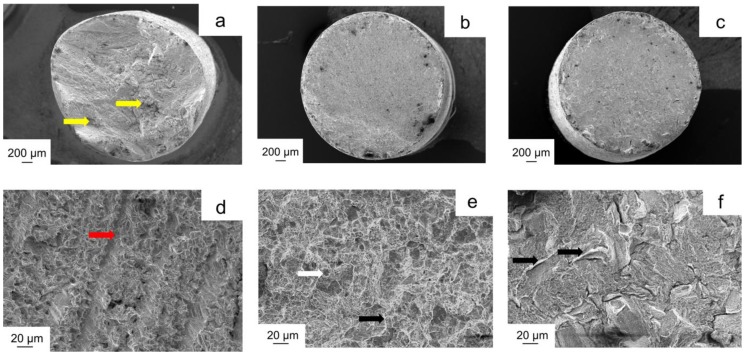
The fractured surfaces of the specimens after the tensile test at 65× magnification: (**a**) casting; (**b**) milling; (**c**) SLM and at 1000× magnification: (**d**) casting; (**e**) milling; and (**f**) SLM. The yellow arrows represent defects; the red arrow represents cleavage planes; the black arrows represent tear ridges, and the white arrow represents dimples.

Figure 4 represents the fracture surfaces of the Co-Cr specimens after the tensile test. The casting group showed a rough fracture surface with many defects (yellow arrows) (Figure 4a). Under high magnification, the surface was wavy with some cleavage planes (red arrow; Figure 4d). The milling group showed a clean and homogenous fracture surface (Figure 4b) with tear ridges and some little dimples (black and white arrows; Figure 4e). For the SLM group, the surface was also homogenous, and tear ridges were observed (Figure 4c,f). 

### 3.3. Bond Strength Test and Microstructure of Metal-Ceramic Specimens

The result of the bond strength is shown in Table 5. The strength value for the casting group was lower than those for the milling and SLM groups (*p* < 0.05), while no significant differences between the milling and SLM groups were found. According to ISO 9693-1: 2012, if the bond strength for more than 66% samples ≥ 25 MPa, the group passes the test [23]. For the casting group, nine samples were lower than 25 MPa, and for the milling and SLM groups, only two samples lower than this level (the total samples in each group were 29, 40, and 40). So all the groups passed the bond strength due to the adequate passing rate.

**Figure 5:**
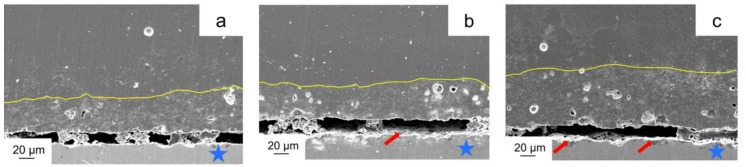
Representative SEM micrograph of the interface of metal-ceramic specimens after the three-point bending test (magnification 1000×): (**a**) casting; (**b**) milling; (**c**) SLM. The yellow lines represent the boundary of the opaque ceramic; the blue asterisks indicate the alloy substrate and the red arrows show the opaque ceramic remaining on the alloy surface.

Figure 5 indicates the cross-section of the metal-ceramic specimens after the three-point bending test. For the casting group, the cracks propagated along the metal-ceramic interface smoothly without adherent ceramic (Figure 5a). While for the milling and SLM groups, cracks spread roughly with some remaining opaque ceramics (red arrows; Figure 5b,c).

**Figure 6:**
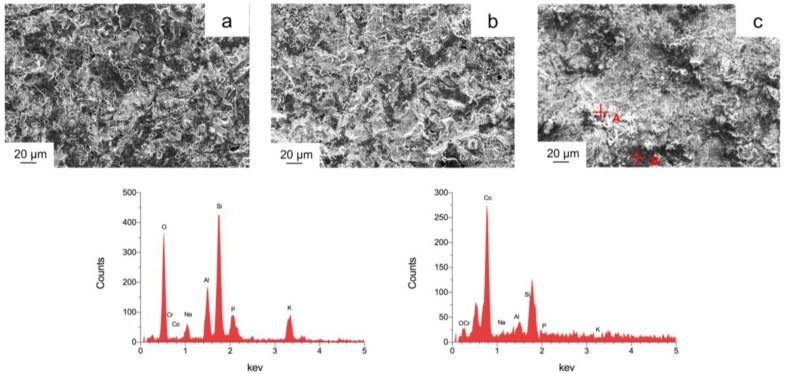
Representative SEM micrographs (magnification 1000×) and EDX images of the Co-Cr alloy substrate surface after debonding from ceramic (**a**) SEM image, casting; (**b**) SEM image, milling; (**c**) SEM image, SLM; (**d**) EDX image, spot A (white area); and (**e**) EDX image, spot B (black area).

Figure 6 shows the Co-Cr substrate surface of the specimens after debonding from the ceramic. After the three-point bending test, the cracked specimens were manually debonded to analyze the remaining ceramic area proportions on the substrate surface. The white areas represent the adherent opaque ceramic on the alloy surface (light color due to its low electrical conductivity), and the black areas indicate the Co–Cr alloy substrate (dark color due to the high conductivity), which was confirmed by spot EDX analysis. White area (spot A) contained mainly the elements Si, O, Al, and K, in contrast, the black area (spot B) consisted mainly of Co, Cr, and a metalloid element (Si). Besides, Figure 6 also indicated that after debonding, the adherent ceramic on the casting alloy surface was less than the milling and SLM groups.

**Figure 7:**
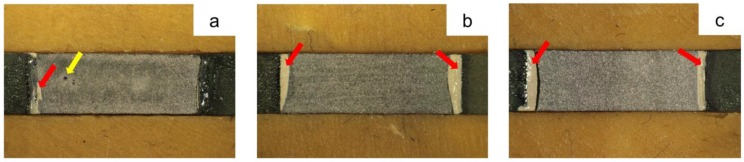
Optical microphotographs of Co-Cr alloy surface after debonding with ceramic (magnification 16×) (**a**) casting; (**b**) milling; and (**c**) SLM. The red arrows represent adherent ceramic, and the yellow arrow represents the pores.

Figure 7 reveals the optical microphotographs of Co-Cr alloy surface after debonding with ceramic. Compared with the casting group, the milling, and SLM groups showed a homogenous appearance without pores. Besides, more adherent ceramic on the alloy surface in milling and SLM groups was observed.

Table 6 shows the frequency of failure mode and the percentage of adherent ceramic area after debonding with ceramic. The failure mode in all groups was adhesive. The residual ceramic on the debonding alloy surface of the specimens was higher in the milling and SLM groups than in the casting group (*p* < 0.05).

## 4. Discussion

In this study, tested specimens of the SLM groups indicated enhanced mechanical properties and comparable metal-ceramic bond strength compared with the casting and milling groups. Therefore, the null hypothesis is rejected.

Since the evaluation of mechanical properties is complex, many standards and experimental techniques have been proposed for different purposes, which are used widely in engineering [40,41,42,43]. For dental restorations, the most commonly used standard is ISO 22674: 2016 [24]. Based on the mechanical properties result of this study (Table 4), the SLM specimens displayed better ductility, toughness, and hardness compared with the casting and milling specimens due to the higher R_p0.2_, R_m_, and Vickers hardness values. For the resistance of fracture, the SLM group showed a higher capacity than the milling group (elongation at break). Besides, the specimens manufactured by milling had the highest rigidity among the three groups because of the higher Young’s modulus values compared to the other groups. This different mechanical performance can be explained by the porosity, grain size, and second-phase particles observed in the respective groups. 

Porosity is an important drawback of casting. In this study, the casting specimens exhibited an inhomogeneous appearance with many pores (Figure 7a), which is associated with the shrinkage and dendritic structure during the solidification of the casting Co-Cr alloy [44]. Differences exist in the solute distribution between the dendritic and interdendritic areas due to the dendritic segregation, and this might reduce the mechanical properties (Figure 3a) [26]. In contrast, the milling and SLM groups showed a homogenous and dense structure without pores (Figure 7b,c). For the milling group, the blanks have been industrially manufactured in an optimized and controlled process, while for the SLM group, complete local melting and rapid solidification can minimize flaws and porosities. This might be an explanation for the superior mechanical properties of the milling and SLM specimens compared to the casting group. 

Additionally, in this study, the grain size of the SLM samples was the smallest, followed by milling and casting. It is a well-known fact that smaller grains are obtained with a fast cooling process such as SLM compared to a slow cooling process like casting [34]. The milling blanks used in this study were made by the powder metallurgy method to obtain an extremely finely grained and 100% homogenous milling blank. As for the SLM powder, only the finest powder with a very narrow grain size distribution was chosen. Zhou et al. and Xin et al. also showed much finer grains and superior mechanical properties of the Co-Cr alloy fabricated by SLM compared to the casting technique [1,26]. In other alloys, different grain size can also lead to different mechanical performance [45]. Grain refinement can strengthen the alloy and improve the ductility and toughness, which may be an important reason for the improved mechanical performance of SLM compared to casting and milling, especially in R_p0.2_, R_m_, elongation, and Vickers hardness. Besides, rapid solidification of the SLM specimens might increase the solution limit of solute elements and reduce dendritic segregation, which would also improve the mechanical performance. The rapid solidification could increase the solution strengthening effects through maintaining a high super saturation, and the supersaturated solid solution element would precipitate later and reinforce the second-phase strengthening effect [26]. This study indicated that the milling and SLM specimens contained dispersed second-phase particles (Figure 3b,c), especially in the SLM group. The second phase particles can improve the deformation resistance of the alloy because the dislocation motion is hindered by the second-phase through forming large dislocation loops, this is the so-called second–phase strengthening effect [36]. Zhou et al. and Jabbari et al. also found the presence of the second-phase microstructure in the milling and SLM specimens [26,46]. 

Besides, in the SLM technique, Co-Cr particles are fused by applying Yb-fiber laser, and objects are built up layer by layer. After completion of the samples, brittle and inhomogeneous objects are obtained due to localized melting. For this reason, a post-build heat treatment process must be applied for the SLM specimens to eliminate the residual stresses, improve mechanical strength and homogeneity, but for casting and milling, this process is not necessary [5,47]. In this study, the SLM group revealed a microstructure with coarse and continuous precipitate after heat treatment (Figure 3c), which implied that furnace cooling provided sufficient time for phase precipitating [48]. Lu et al. indicated that the expected mechanical properties of SLM manufactured Co-Cr alloys could be tailored by recrystallization under the optimal heat treatment conditions, which is affected by the heating and cooling rate, holding time, cooling media, etc. [48]. Additionally, the lower mechanical properties of the casting groups could be supported by the morphology of the fracture surface. The casting group showed a defected appearance with some cleavage planes, which indicated a typical brittle fracture behavior with poor ductility (Figure 4). For the milling and SLM specimens, the fracture surfaces are dense and free of large internal defects with some tear ridges, indicating a highly ductile fracture [48].

The bond strength for the casting group was lower than those for the milling and SLM groups (*p* < 0.05, Table 5), indicating an easier debonding. The different bond strength can be explained by Young’s modulus, chemical bonding, mechanical interlocking, and compressive bonding. 

Normally, an alloy with higher Young’s modulus could be more rigid to resist bending and delamination, thus leading to stronger metal-ceramic bond strength. In this study, the casting group revealed the lowest bond strength between alloy and ceramic, which might be partially attributed to the lowest rigidity. Besides, in the calculation of bond strength (Equation (1)), correction factors, A, B, and C are calculated by the Young’s modulus of the tensile test. Therefore, the Young’s modulus values would influence the bond strength result, even though the failure forces for each group were different.

Chemical bonding is a result of chemisorption by diffusion in the metal-ceramic interface [6], which is influenced by the oxide layer between metal and ceramic. In this study, the different bond strength among groups might relate to the different thickness of the oxide layer. Wang et al. reported a lower metal-ceramic bond strength and a thicker oxide layer between alloy and ceramic for the casted substrates compared with milling and SLM [36]. Akova et al. and Serra-Prat et al. also reported different manufacturing methods would lead to different oxide layer thickness [38,39]. These findings are in accordance with our assumption. The oxidation rates among different manufacturing processes should be further examined, since the oxide layer may influence the chemical bonding, and thus, the metal-ceramic bond strength.

Mechanical interlocking is another parameter for bond strength. A general consideration is that higher roughness would lead to increased metal-ceramic bond strength due to the improved contact area and mechanical interlocking on the metal-ceramic interface [49,50]. This might be an explanation for why the milled samples showed a higher bond strength compared to the casting group (Table 3). However, extensive roughness may reduce the bond between alloy and ceramic, and this might explain why the highest R_a_ value of the SLM substrates did not represent the strongest bond [51]. The relatively higher roughness of the SLM group could be related to the adherence of partially melted powder particles on the alloy surface. Fox et al. studied the effect of process parameters on the surface roughness of SLM fabricated samples and revealed a large number of the partially melted powder particles on the alloy surfaces, which might affect the roughness [52]. The rougher surface of the SLM specimen surface is also related to the partial fusion of isolated powder particles during fabricating (balling phenomenon) [53]. Balling is a defect that appears when the SLM process parameters are not correctly set, and the molten tracks break into separate balls due to the surface tension effect. The SLM powder particles are loosely attached to the metal substrate on the borderline of the laser fusion, then leading to a rough surface [54]. However, the surface roughness of SLM substrates is also influenced by the manufacturing parameters, such as the powder particle size, material composition, layer thickness, geometry of the object, building direction, etc. [54]. Besides, the SLM specimens showed a better bond strength than the casting specimens, which may also be related to the gap between layers caused by the SLM manufacturing process. The gap may widen the surface area, and the bond strength may improve because of the penetration of the ceramic through the gaps. Our assumption is supported by a previous study. Wu et al. found an intermediate layer and elemental interpenetration in the metal-ceramic interface of the SLM alloy [25].

Compressive bonding at the metal-ceramic interface is also important for the metal-ceramic bond strength. Ideally, the CTE of the metal should be slightly higher than the ceramic since a compressive strength may occur in the ceramic layer during the cooling stage after firing, this is what is called a positive CTE mismatch [34]. However, the CTE values of the Co-Cr alloy and ceramic in this study were 14.1 × 10^−6^ K^−1^ and 13.9–15.1 ×10^−6^ K^−1^ (Table 1), they were not expected because the value of the alloy was lower than the upper boundary value of the ceramic. The non-ideal CTE values between alloy and ceramic may be a reason for the low adherent ceramic on the alloy surface after debonding. The failure mode among all three groups was adhesive (Table 6), implying that the crack occurred between the metal and ceramic (Figure 5 and Figure 7). Usually, adhesive failure is not an ideal situation, because this indicates a lower bond between the metal and ceramic than that within the ceramic interior and fewer destructive forces were needed to separate them [55]. 

Summed up, our results indicate that according to ISO 22674: 2016 and ISO 9693-1: 2012, the SLM technique is suitable to be used with the Co-Cr alloy for fixed dental restorations comprising crowns and bridges, due to the superior mechanical properties and adequate metal-ceramic bond strength. The casted samples could also be used for crowns and bridges, but before clinical applications, the objects should be tested first due to the relatively low performance in metal-ceramic bond strength (the passing rate for the casting group in three-point bending test is 69%, very close to the required rate of 66% by the ISO standard). The milling technique could only be recommended for dental bridges because the elongation at fracture did not pass the requirement for dental crowns, even though the milled samples showed a good performance in other mechanical properties and metal-ceramic bond strength. Compared with the casting and milling techniques, SLM might be a superior technique for the manufacturing of fixed dental restorations for several reasons. First, SLM can fabricate complex samples without apparent waste. Second, technician error can be reduced, and the quality of restorations increased because of the enhanced productivity. Third, the manufacturing costs of restoration might be decreased through large-scale production. Fourth, patients could benefit from shorter production cycles.

## 5. Conclusions

Based on the materials used and the limitations of this study, the following conclusions can be drawn:

1. The Co-Cr alloy specimens manufactured by the SLM technique have enhanced mechanical properties and comparable metal-ceramic bond strength compared with the specimens prepared by the casting and milling techniques;
2. The microstructure of the Co-Cr alloy depends on the manufacturing techniques. Compared with casting and milling, the SLM specimens have relatively homogeneously distributed fine grains and more dispersed second-phase particles;
3. According to the ISO 22674: 2016 and ISO 9693-1: 2012, the SLM technique can be used for fabricating dental bridges and crowns. SLM might be superior to the traditional manufacturing techniques for fixed dental restorations.

## Figures and Tables

**Table 1:** Specification of the alloy and ceramic used in this study.

Materials	Brand Name	Material Type	Composition (wt %)	CTE (×10^−6^K^−1^)	Manufacturer
Co-Cr Alloy	Remanium Star	Metal ingots: casting	Co 60.5%, Cr 28%, W 9%, Si 1.5%, Other (Mn, N, Nb, Fe) < 1%	14.1	Dentaurum, Ispringen, Germany
	Remanium Star MDI	Metal blank: milling		14.1	
	Remanium Star CL	Metal powder: SLM		14.1	
Ceramic	Ceramotion Me	Dental ceramic	Glass (silica) based ceramic	13.9–15.1	

CTE: coefficient of thermal expansion.

**Table 2:** Firing schedules of the procedure for veneering ceramic.

Product Name	Pre-Heating Temp. (°C)	Drying Time (min)	Heating Rate (°C/min)	Final Temp. (°C)	Holding Time (min)	Vacuum
Paste opaque	500	8	75	950	1	+
1st Dentin	500	6	55	870	2	+
2nd Dentin	500	6	55	870	1	+
Glaze	500	6	75	860	1	-

**Table 3:** Surface roughness (means ± standard deviations) of the test groups.

Group	R_a_ (μm)
Casting	1.27 ± 0.10 ^a^
Milling	1.33 ± 0.10 ^a^
SLM	1.51 ± 0.58 ^b^

Different lowercase letters in the same column indicate significantly different groups (*p* < 0.05).

**Table 4:** Mechanical properties (means ± standard deviations) of the casting, milling, and SLM groups.

Group	R_p0.2_ (MPa)	R_m_ (MPa)	Elongation (%)	Young’s Modulus (GPa)	Hardness (Hv 10)
Casting	581 ± 16 ^a^	783 ± 32 ^a^	12 ± 2 ^ab^	188 ± 19 ^a^	303 ± 15 ^a^
Milling	672 ± 4 ^b^	1069 ± 10 ^b^	10 ± 1 ^a^	253 ± 14 ^b^	353 ± 6 ^b^
SLM	783 ± 15 ^c^	1158 ± 10 ^c^	13 ± 1 ^b^	195 ± 15 ^a^	399 ± 24 ^c^

Different lowercase letters in the same column indicate significantly different groups (*p* < 0.05).

**Table 5:** Metal-ceramic bond strength (means ± standard deviations) of the casting, milling, and SLM groups.

Group	Bond Strength (MPa)	Passing Rate (%)
Casting	26.86 ± 3.48 ^a^	69%
Milling	34.22 ± 7.77 ^b^	95%
SLM	29.88 ± 3.62 ^b^	95%

Different lowercase letters in the same column indicate significantly different groups (*p* < 0.05).

**Table 6:** The failure mode analysis and the area fracture of adherence ceramic.

Group	Frequency of the Failure Mode (*n*)			Area Fraction of Adhering Ceramic (%)
	Adhesive	Cohesive	Mixed	
Casting	29	0	0	5.5 ± 3.1 ^a^
Milling	40	0	0	7.9 ± 3.4 ^b^
SLM	40	0	0	8.0 ± 3.2 ^b^

Different lowercase letters in the same column indicate significantly different groups (*p* < 0.05).

## Abbreviations

ANOVA	One-way analysis of variance
BSE	Backscattered electrons
CAD-CAM	Computer-aided design and computer-aided manufacturing
Co-Cr	Cobalt-chromium
CTE	Coefficient of thermal expansion
EDX	Energy dispersive X-Ray spectroscopy
PFM	Porcelain-fused-to-metal
R_a_	Arithmetic average of the roughness profile
R_m_	Ultimate tensile strength
R_p0.2_	0.2 % yield strength
SEM	Scanning electron microscopy
SLM	Selective laser melting
